# First Experience With the Utility of ReMAP (Repeatable Microcatheter Access Port) in Portal Vein Stenting

**DOI:** 10.7759/cureus.58530

**Published:** 2024-04-18

**Authors:** Kentaro Fujimoto, Takayuki Kondo, Hidemi Unozawa, Jun Koizumi, Naoya Kato

**Affiliations:** 1 Gastroenterology, Chiba University, Chiba, JPN; 2 Radiology, Chiba University, Chiba, JPN

**Keywords:** endoscopic injection sclerotherapy(eis), esophageal variceal bleed, extrahepatic portal vein stenosis(ehpvs), extrahepatic portal vein obstruction (ehpvo), transjugular intrahepatic portosystemic shunt (tips), remap

## Abstract

Portal vein stenting is a treatment option for portal hypertension caused by extrahepatic portal vein obstruction or stenosis. However, limited pathways to approach the portal vein are available, hindering re-intervention in the portal vein. Portal vein puncture through the transjugular intrahepatic portosystemic shunt route is less invasive and considered suitable for portal vein stenting. Furthermore, transjugular intrahepatic portosystemic shunting facilitates repeat approaches to the portal vein. However, a transjugular intrahepatic portosystemic shunt stent is not recommended unless necessary because of adverse events, and cannot be retrieved, once placed. Herein, we report on a novel approach using the repeatable microcatheter access port: ReMAP™ (Toray, Tokyo, Japan), a central vein port into which a 2.9 Fr catheter can be inserted. We used it for a repeat approach to the portal vein with only one puncture and without placing a transjugular intrahepatic portosystemic shunt stent.

## Introduction

Extrahepatic portal vein obstruction (EHPVO) or stenosis (EHPVS) causes portal hypertension and accounts for 5-10% of all cases of portal hypertension [1,2]. Moreover, EHPVO or EHPVS is caused by pancreatitis, appendicitis, tumors, and postsurgical adhesive portal venous stenosis [3,4]. Portal vein stenting is a treatment option for portal hypertension caused by EHPVO or EHPVS [5]. Portal vein stenting is performed via a percutaneous transhepatic approach, laparotomy via the transileocolic vein, or a transjugular intrahepatic portosystemic route [6,7]. Additionally, liver abscesses, intraperitoneal bleeding, bile duct injuries, and biliary bleeding have been reported using the percutaneous transhepatic approach [5]. Laparotomy via the transileocolic vein is relatively invasive. Although portal vein puncture via a transjugular intrahepatic portosystemic shunt (TIPS) is difficult, it is less invasive and is suitable for portal vein stenting [8]. However, limited pathways are available to approach the portal vein, hindering re-intervention in the portal vein.

Furthermore, TIPS stenting facilitates a repeatable approach to the portal vein but may cause cirrhotic cardiomyopathy, hepatic encephalopathy, and decreased hepatic blood flow. A TIPS stent cannot be retrieved once placed. Therefore, TIPS stent placement is not recommended unless necessary [8]. However, there have been reports of early occlusion of portal vein stents owing to portal vein thrombosis [6], which may require additional treatment and a route to the portal vein must be secured after portal vein stenting.

To our knowledge, no method that allows repeated approaches to the portal vein has been reported. Herein, we report a novel portal vein approach using the repeatable microcatheter access port: ReMAP™ (Toray, Tokyo, Japan), a central vein port into which a catheter can be inserted.

## Case presentation

**Figure 1:**
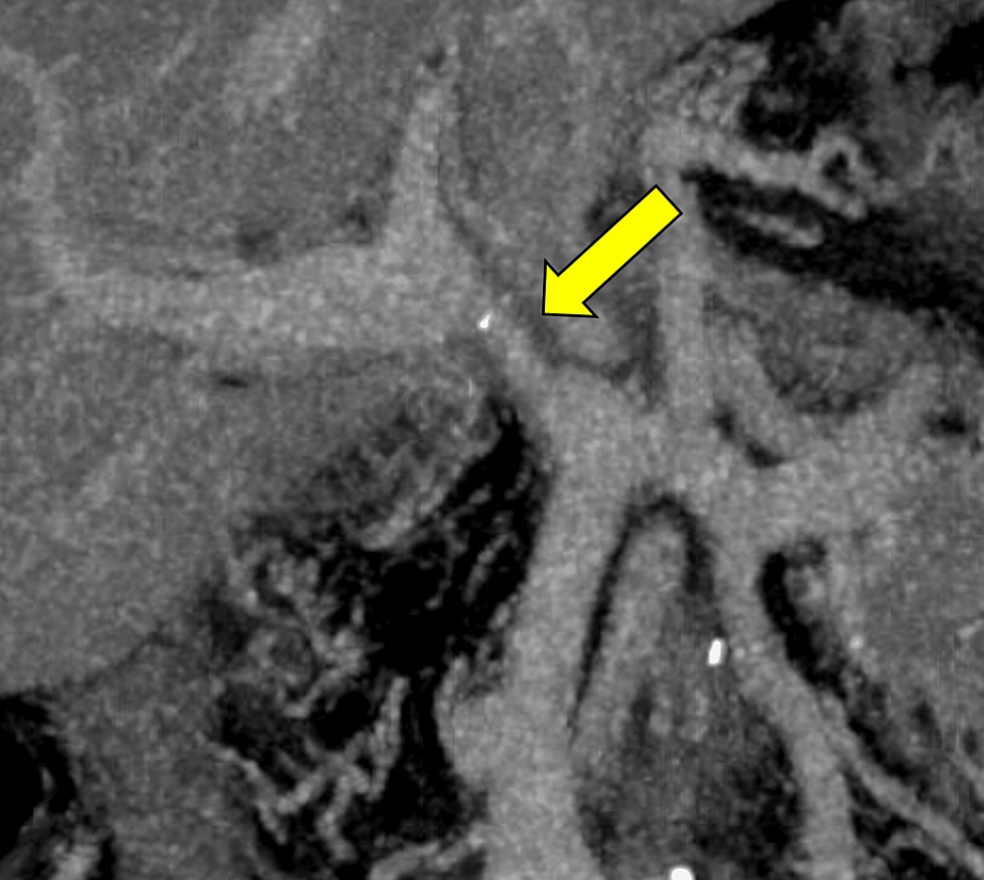
CECT reformatted coronal section. Stenosis of the extrahepatic portal vein before dilation (yellow arrow). CECT: Contrast-enhanced computed tomography.

The patient was a 43-year-old man who had undergone pancreatoduodenectomy for an inflammatory pseudotumor in the duodenum at another hospital. Two years later, esophageal varices formed, and endoscopic variceal ligation was performed. However, four years later, the patient was referred to our hospital for treatment of recurrent esophageal varices. Triphasic computed tomography revealed extrahepatic portal vein stenosis (Figure 1).

**Figure 2:**
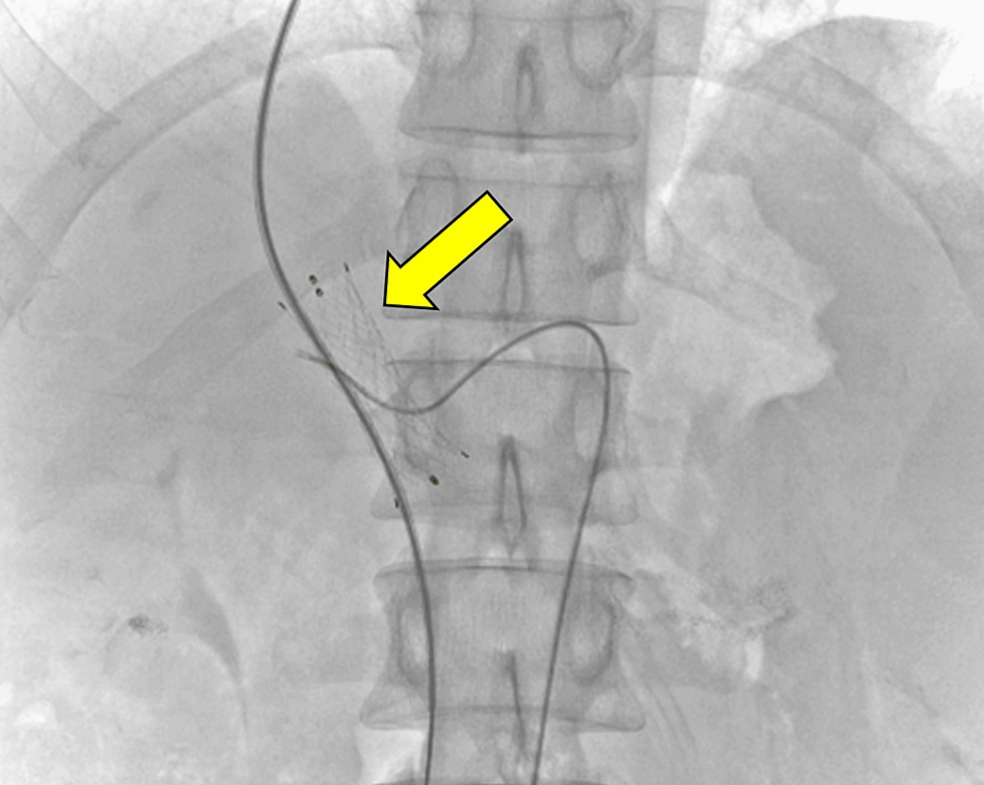
Angiographic image. Portal vein stenting using a 12 × 40 mm E-Luminexx™ stent (yellow arrow). E-Luminexx™ (C.R. Bard Inc, Tempe, AZ, USA).

Furthermore, endoscopic sclerotherapy for esophageal varices was successfully administered at our hospital. However, recurrence of esophageal varices was considered highly probable owing to residual EHPVS. Therefore, we planned to implant a portal stent in the EHPVS as a long-term treatment strategy. The right internal jugular vein was punctured, a 0.035-inch 180 cm guide wire was inserted into an 8 Fr 50 cm sheath, and a 5.2 Fr balloon catheter was introduced. We punctured the right branch of the portal vein (P8) from the right hepatic vein using the Rösch-Uchida Transjugular Liver Access Set (Cook Medical, Tokyo, Japan). The stenosis was 26 mm long, with a diameter of 4.6 mm. Balloon dilation of the stenosis was performed using a 12 × 40 mm over-the-wire angioplasty balloon. After dilation, a 12 × 40 mm uncovered stent was placed (Figure 2).

**Figure 3:**
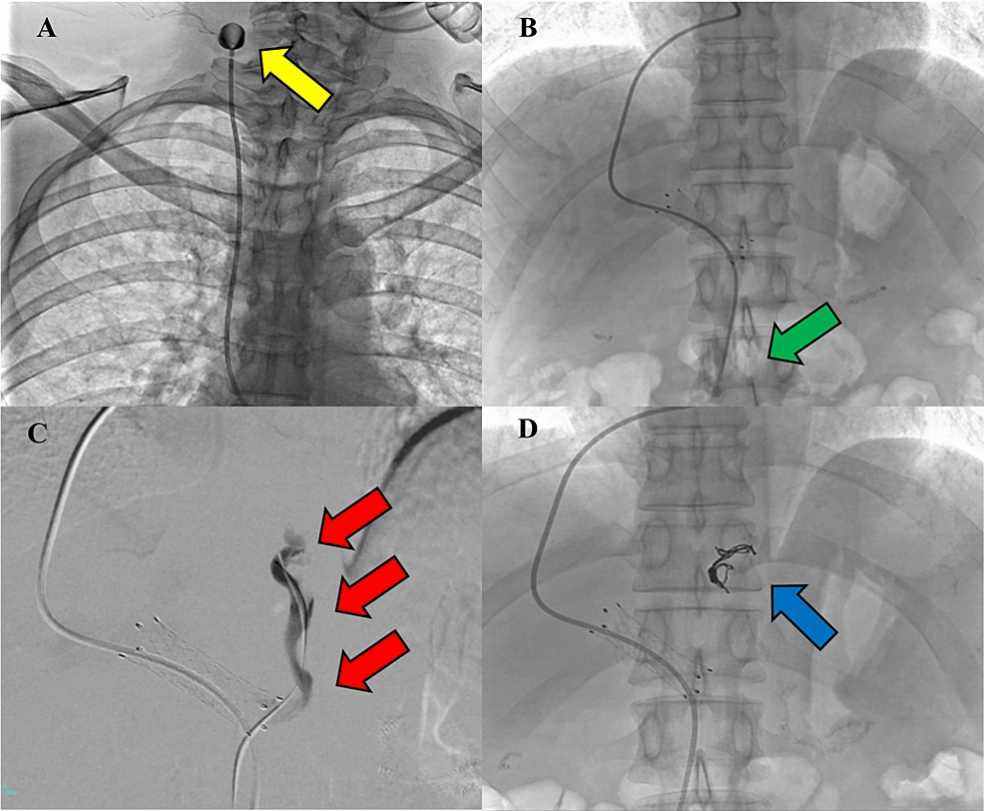
(A, B) Angiographic image. ReMAP™ placement. ReMAP™ was placed in the right jugular vein (yellow arrow) and the catheter tip was placed in the superior mesenteric vein (green arrow). (C, D) Angiographic image. Coil embolization on the left gastric vein using 6 × 200 mm Target XL™ 360 soft coils and 8 × 300 mm Target XL™ 360 soft coils. Left gastric vein angiography (red arrow), and coil embolization fluoroscopic image (blue arrow). ReMAP™ (Toray, Tokyo, Japan), Target XL™ (Stryker Neurovascular, Fremont, CA, USA).

After stenting, the portal pressure decreased from 30 mmHg to 15 mmHg. The portal vein diameter increased from 4.6 mm to 9.0 mm. Subsequently, the catheter tip was placed in the superior mesenteric vein (Figure 3, panel B), and the ReMAP™ was placed in the right jugular vein via the right internal jugular vein (Figure 3, panel A). One week later, a 2.9 Fr 110 cm microcatheter was inserted from the ReMAP™ to evaluate the stent. Moreover, the stent was patent, and no thrombi were observed. However, residual distal hepatic blood flow was observed in the left gastric vein (Figure 3, panel C). Coil embolization was performed on the left gastric vein using a 1.6 Fr Carnelian® MARVEL S microcatheter (Tokai Medical Products, Aichi, Japan) to prevent the recurrence of the esophageal varices (Figure 3, panel D). We used 6 × 200 mm soft coils and 8 × 300 mm soft coils for coil embolization. Immediately after coil embolization, the ReMAP™ and catheter were quickly removed. Six months after portal vein stenting, the stent remained patent, with no recurrence of esophageal varices.

## Discussion

To our knowledge, this is the first report of a two-stage treatment strategy for portal vein obstruction using ReMAP™ instead of a TIPS stent. Several studies reported the effectiveness of portal vein stent placement for portal hypertension caused by benign main portal vein stenosis [5,8]. Yamakado et al. found that portal venous stent placement decreases portal venous blood pressure [4]. Yunghun et al. also reported that portal venous stenting is effective in stopping variceal bleeding as well as alleviating refractory ascites by lowering portal pressures [9].

**Figure 4:**
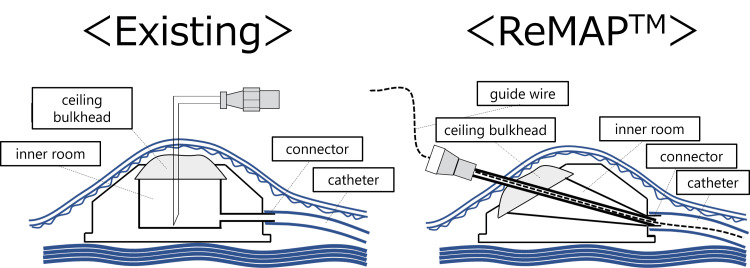
Structural differences between conventional CV ports and ReMAP™. With conventional CV ports, only puncture is possible; with ReMAP™, catheter insertion is possible. CV: Central venous. ReMAP™ (Toray, Tokyo, Japan).

ReMAP™ was originally used for arterial infusion therapy; however, in this case, it was used for a repeat approach to the portal vein with only one puncture and without the placement of a TIPS stent. Additionally, with ReMAP™, 2.9 Fr catheter-based coil embolization is feasible. A 0.035-inch guidewire can be also inserted through the ReMAP™ system (Figure 4).

Therefore, a catheter of 6 Fr or more can be inserted, and other treatments, such as balloon dilation or additional portal vein stenting, are also possible after the remaining ReMAP™ is withdrawn. Moreover, ReMAP™ is available for thrombectomy and drug administration to the portal vein. In the future, ReMAP™ may contribute to the diversification of treatment administration to the portal vein. However, since only microcatheters can be directly inserted with ReMAP™, if the catheter diameter that needs to be inserted for treatment is too large, the ReMAP™ must be removed, although catheter replacement with a guidewire is possible. In addition, there are complications similar to those of conventional CV ports, such as thrombosis and catheter infection. There is no fixed period of time for long-term use, and since the possibility of complications increases, prompt removal is recommended when removal is possible. Since this case involved a short-term implantation, further study is needed regarding long-term use.

## Conclusions

Portal vein stenting is an effective treatment for extrahepatic portal vein obstruction. However, re-approaching the portal vein for additional treatment or re-treatment is difficult. ReMAP™ is a central venous port into which a catheter can be inserted, allowing easy re-approach to the portal vein without the complications of transjugular intrahepatic portosystemic shunt stenting. Thus, in this case, we used ReMAP™ for repeated approaches to the portal vein. Therefore, we propose the use of ReMAP™ as a novel treatment option for the portal vein in the future.
